# Potential impact of the COVID-19 pandemic on japanese patients with eating disorders -a cross-sectional study

**DOI:** 10.1186/s13030-021-00232-z

**Published:** 2022-01-06

**Authors:** Shu Takakura, Kenta Toda, Makoto Yamashita, Tomoko Kitajima, Takafumi Suematsu, Hiroaki Yokoyama, Chie Suzuyama Asou, Tomokazu Hata, Nobuyuki Sudo

**Affiliations:** 1 Department of Psychosomatic Medicine, Kyushu University Hospital, Fukuoka, Japan; 2 Fukuoka Prefectural Support Base Hospital for Eating Disorders, Fukuoka, Japan; 3 Department of Psychosomatic Medicine, Graduate School of Medical Sciences, Kyushu University, 3-1-1 Maidashi, Higashi-ku, 812-8582 Fukuoka, Japan

**Keywords:** Eating disorders, COVID-19, Eating disorder inventory, Parental bonding instrument, Maternal care

## Abstract

**Background:**

The COVID-19 pandemic and associated lockdown had a considerable impact on eating disorders (EDs). We evaluated the clinical features of Japanese ED patients before and after the first COVID-19 outbreak-related state of emergency (April 7, 2020).

**Methods:**

We studied 148 patients who were divided into two groups based on when they arrived at our clinic: before (Before group: *n* = 86) or after (After group: *n* = 62) the start of the first state of emergency. All patients completed the Japanese versions of the Eating Disorder Inventory (EDI) and Parental Bonding Instrument (PBI).

**Results:**

The After group was substantially younger than the Before group (*p* = .0187). Regardless of the ED type, patients who developed an ED during the first state of emergency tended to be significantly younger than those who developed one before. Differences in EDI characteristics were observed between the two groups. The PBI care subscale was notably higher (*p* = .0177) in the After group. The PBI maternal care subscale was the only statistically significant factor associated with age (β = -0.35, *p* < .0001).

**Conclusions:**

Home confinement associated with the COVID-19 pandemic and the ensuing increase in parent-child closeness may have influenced the decreased age of ED patients at their initial consultation. Treatment interventions should consider the differences in the clinical features of EDs.

## Background

Eating disorders (EDs) are closely linked to psychosocial factors in their onset and course, and they become more severe when prolonged [1]. According to reports, the COVID-19 pandemic in late 2019 and the associated lockdown had a detrimental impact on EDs [2–9]. In addition to the psychosocial stress induced by pandemics, stay-at-home orders may exacerbate EDs [10]. Indeed, stress induced by lockdowns has been associated with binge eating and dietary restrictions, leading to dietary problems among general students [11] and students with dietary concerns [12]. Further, reports on the effects of the COVID-19 pandemic on EDs in Japan have yet to be made.

On April 7, 2020, Japan declared a state of emergency in seven prefectures, which was later expanded nationwide on April 16. Schools were closed nationwide, forcing students to remain at home. This is the most stringent state of emergency order issued to date, and subsequent orders have not resulted in school closures. The schools in Fukuoka Prefecture, where the majority of the current study’s participants lived, were closed from April 7 to May 14, 2020. In our clinic, the total number of patients with EDs decreased during the state of emergency and later increased. Furthermore, the number of younger patients with EDs has been increasing since May 14, 2020. Given that the COVID-19 pandemic may have influenced the onset and course of EDs in Japan [2–9], the following hypotheses were proposed. First, patients with EDs after the first state of emergency would be more likely to be younger than those diagnosed before. Second, many young patients may have developed EDs during the first state of emergency. Third, there may be differences in the proportion of disease classifications among ED patients before and after the first state of emergency. Fourth, the psychological characteristics of ED patients before and after the first state of emergency may differ. Based on these hypotheses, the current study sought to investigate the characteristics of ED patients before and after the first state of emergency.

## Methods

### Participants and clinical data

Data were extracted from the medical records of 186 Japanese female ED patients who first visited the Department of Psychosomatic Medicine at Kyushu University Hospital between April 1, 2019, and March 31, 2021. The hospital provides specialized treatment for EDs, and more than 500 patients with a variety of psychosomatic diseases visit every year. They were diagnosed with an ED according to the Diagnostic and Statistical Manual of Mental Disorders, Fifth Edition. Excluded were 38 patients with missing data. The remaining 148 patients were divided into two groups based on the timing of their visit to our clinic: Before group, before the onset of the first state of emergency (before April 7, 2020) (*n* = 86); After group, during and after the onset of the first state of emergency (*n* = 62). Instead of obtaining informed consent from the participants, information on study implementation, including the nature and purpose of the study, was released on the website of the Department of Psychosomatic Medicine, Kyushu University. This ensured ample opportunity for patients to decline to participate. This study received approval from the Kyushu University Research Ethics Committee (No. 28-20).

### Psychological parameters

During their first visit to the clinic, all patients completed the Japanese versions of the Eating Disorder Inventory (EDI) [13] to measure their ED pathology and the Parental Bonding Instrument (PBI) [14] to assess their relationship with their mothers. The EDI is a self-report questionnaire that comprises 64 items and consists of eight subscales: *drive for thinness, lack of interoceptive awareness, bulimia, body dissatisfaction, ineffectiveness, maturity fears, perfectionism*, and *interpersonal distrust*. Each question of the EDI features a 6-point scale ranging from “never” to “always”, with scores ranging from 0 to 3 points. The PBI is a self-report questionnaire that comprises 25 items consisting of two subscales evaluating parental bonding style from a child’s perspective: *care* and *over-protection*. Each question of the PBI features a 5-point scale ranging from “very unlike” to “very like”, with scores ranging from 0 to 3 points. Both instruments have been standardized and validated for use with Japanese patients [15, 16].

### Data analysis

We employed the Wilcoxon rank sum test to compare the two groups after testing for a normal distribution using the Shapiro–Wilk test. Following the Kruskal–Wallis tests, we conducted Steel–Dwass tests for multiple comparisons. The chi-square test was used to analyze categorical data. Next, we ran stepwise multiple regression analyses to identify age predictors.

All tests were considered statistically significant at *p* < .05. All statistical analyses were performed using JMP^®^ version 16 for Mac OS (SAS Institute Inc., Cary NC, 1989-2021).

## Results

### Clinical characteristics

**Fig. 1:**
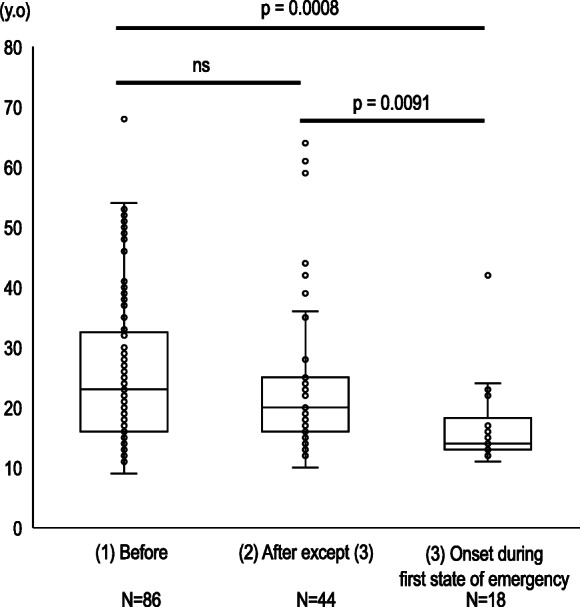
Box plot of the age of the patients who visited our clinic (1) before or (2) after the first state of emergency or (3) who developed an ED during the first state of emergency. (2) is a group of patients who visited our clinic after the first state of emergency was declared, excluding the patients who developed their ED during the first state of emergency

**Fig. 2:**
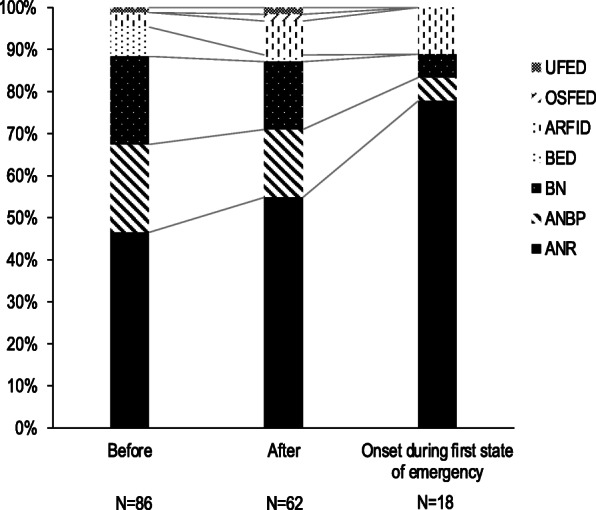
Proportion of eating disorders by diagnosis of the Before group, the After group, and the group of patients who developed an ED during the first state of emergency. AN: anorexia nervosa, ANR: restricting type of anorexia nervosa, ANBP: binge eating / purging type of anorexia nervosa, BN: bulimia nervosa, ARFID: avoidant / restrictive food intake disorder, BED: binge eating disorder, OSFED: other specified feeding and eating disorder, UFED: unspecified feeding or eating disorder

Data from 148 of the 186 patients were analyzed. Table 1 shows the clinical characteristics of each group. Age was significantly lower in the After group than in the Before group (19.0 vs. 22.0 years, *p* = .0187). We found no significant difference in age between the two groups when we excluded patients who developed some variation of ED during the first state of emergency in the After group (20.0 vs. 22.0 years old, *p* = .6458) (Fig. 1). Patients who developed an ED during the first state of emergency were significantly younger than those of the Before group (14.0 vs. 22.0 years, *p* = .0008) and After group that excluded patients who developed an ED during the first state of emergency (14.0 vs. 20.0 years, *p* = .0091) (Fig. 1). Patients in the After group had a significantly shorter illness duration than those of the Before group (1.4 vs. 3.5 years, *p* = .0049). In contrast, the age of disease onset did not differ between the two groups (16.5 vs. 15.5 years, *p* = .1000). Moreover, no significant difference in body mass index was observed between the two groups (15.3 vs. 15.1, *p* = .3160). Regarding the proportions of ED types, we found that the After group had higher rates of the restricting type of AN (AN-R) (54.8% vs. 46.5%) and avoidant/restrictive food intake disorder (8.1% vs. 3.5%), and a slightly lower rate of the binge eating/purging type of AN (18.9% vs. 20.9%). Also, we discovered an extremely high rate of AN-R among patients who developed an ED during the first state of emergency. The chi-square test revealed that the distribution of AN-R among patients who developed an ED during the first state of emergency was significantly different (77.8%, χ^2^ = 2.78, *p* = .0420) comparing the Before (46.5%, χ^2^ = 0.21) and After group, excluding patients who developed an ED during the first state of emergency (45.5%, χ^2^ = 0.18) (Fig. 2).

**Table 1:** Clinical characteristics of the two goups

	Before		After		p
n	86		62		
	median	(range)	median	(range)	
Age (year)	22.5	(11.0 – 68.0)	19.0	(10.0 – 64.0)	0.0187
Duration of illness (year)	3.5	(0.1 - 48.2)	1.4	(0.1 - 25.7)	0.0049
Age of onset	16.5	(14.0 – 21.0)	15.5	(11.0 – 19.25)	n.s.
BMI (kg/m^2^)	15.3	(10.0 - 42.3)	15.1	(10.1 - 29.1)	n.s.
ED diagnosis					
	n	percentage	n	percentage	
AN					
restricting	40	46.5	34	54.8	
binge eating / purging	18	20.9	10	18.9	
BN	18	20.9	10	16.1	
ARFID	3	3.5	5	8.1	
BED	6	7.0	1	1.6	
OSFED	0	0	1	1.6	
UFED	1	1.2	1	1.4	

ED: eating disorder, AN: anorexia nervosa, BN: bulimia nervosa, ARFID: avoidant / restrictive food intake disorder, OSFED: other specified feeding or eating disorder, UFED: unspecified feeding or eating disorder, range: interquartile range

### Psychological features

Table 2 depicts the psychological features of the two groups. Regarding the EDI subscales, the bulimia subscale was significantly lower in the After group (*p* = .0291). This group also scored significantly lower on the ineffectiveness subscale (*p* = .0443) and significantly higher on the maturity fears subscale (*p* = .0439). Meanwhile, this group also reported lower tendencies in the subscales of drive for thinness and lack of interoceptive awareness (*p* = .0580 and *p* = .0510, respectively). The PBI total score was significantly higher in the After group (*p* = .006), particularly the care subscale score (*p* = .0177).

**Table 2:** Psychological features of the two groups

	Before		After		
n	86		62		
	median	(range)	median	(range)	p
EDI					
Drive for thinness	14.5	(3.8-18.0)	9.0	(2.9-15.5)	0.0580
Lack of interoceptive awareness	13.0	(3.8-23.0)	7.0	(3.0-21.4)	0.0510
Bulimia	4.5	(0-17.0)	1.0	(0-12.0)	0.0291
Body dissatisfaction	14.5	(8.0-22.0)	11.0	(8.0-16.5)	n.s.
Ineffectiveness	11.5	(6.8-19.3)	9.0	(5.0-21.0)	0.0443
Maturity fears	7.0	(5.0-14.3)	9.0	(6.0-18.0)	0.0439
Perfectionism	2.0	(1.0-9.0)	4.0	(1.0-10.7)	n.s.
Interpersonal distrust	8.0	(5.0-14.0)	6.0	(3.0-14)	n.s.
Total	76.5	(44.8-115.0)	58.0	(41.3-96.3)	ns
PBI					
Care	27.0	(19.8-34.0)	31.5	(22.8-36.0)	0.0177
Overprotection	10.0	(4.0-21.3)	9.0	(5.0-20.0)	ns
Total	37.0	(33.0-43.0)	39.5	(36.0-43.9)	0.006

EDI, Eating disorder inventory: PBI, Parental bonding instrument: range, interquartile range

### Factors associated with age

We conducted multiple regression analyses to identify significant factors related to patient age (Table 3). In addition to the presence or absence of ED onset during the first state of emergency, we included significant variables, such as EDI and PBI subscales, as explanatory variables. The care subscale of PBI was the only statistically significant factor associated with age (β = -0.35, 95% CI [- 0.76, - 0.27], *p* <.0001).

**Table 3:** Factors associated with age

	β	t	[95% CI]	p
EDI				
Bulimia	-0.03	0.38	[-0.23, 0.34]	0.70
Ineffectiveness	-0.04	-0.36	[-0.38, 0.26]	0.71
Maturity fear	-0.14	-1.69	[-0.81, 0.06]	0.09
PBI				
Care	-0.35	-4.11	[-0.76, -0.27]	< 0.0001
Onset during first state of emergency	0.14	1.79	[-0.29, 5.80]	0.07

EDI, Eating disorder inventory: PBI, Parental bonding instrument: CI, confidence interval

## Discussion

This is the first report that we are aware of on the impact of the COVID-19 outbreak on Japanese patients with EDs. This study revealed distinct characteristic variations in ED patients before and after Japan’s first state of emergency order that was declared in response to the COVID-19 pandemic. Patients with EDs who first visited the clinic after the initial state of emergency were significantly younger, and the median age of patients who developed an ED during this time was much younger (14 years). However, the age of onset did not differ. Regarding ED pathology, differential clinical features were observed before and after the first state of emergency. Moreover, the maternal care subscale of PBI was the only factor associated with age, based on the significantly high scores recorded.

There are several possible explanations for why a higher number of younger patients were observed in our clinic after the first state of emergency. In the current study, when we compared the patients’ age before and after the first state of emergency, excluding those who developed the disease during the first state of emergency, the age difference receded. Although causality cannot be determined in the current study, this finding demonstrates that patients who developed the disease during the initial state of emergency were remarkably young and suggests that the first state of emergency may have triggered symptoms in younger patients. Stress associated with the COVID-19 outbreak lockdown has been linked to students developing EDs, including binge eating and dietary restrictions [12]. The difference between the first state of emergency and those declared subsequently in Japan was that the former shut down all schools across the country, drastically reducing students’ contact with the outside world. This lack of interaction may have added to the students’ stress and induced feelings of isolation, loneliness, depression, or anxiety during the first state of emergency [17–19]. These feelings of stress and mood changes may act as triggers for students’ eating problems [17, 18]. Moreover, many patients who contracted the disease during the first state of emergency reported feeling fat during the stay-at-home order as a result of reduced contact with the outside world and decreased engagement in exercise and other physical activities.

Meanwhile, poor maternal care has been widely reported to be a risk factor for problematic eating behavior in younger people [20, 21]. The current study found that the level of maternal care was significantly higher in patients who visited the clinic after the first state of emergency. Nearly 80% of the patients who developed EDs during the first state of emergency had AN-R within one year of the onset of their ED, resulting in an early clinic visit. This tendency could be linked to the high level of maternal care discovered in this study. In other words, because of their confinement at home or reduced contact with the outside world during the COVID-19 outbreak, the relationship between mothers and their children may have become stronger and closer, giving mothers more opportunity to observe children’s eating behavior or being more ready to seek help during this period of heightened concern about health. Therefore, it is possible that mothers who had high care noticed the eating behaviors sooner in their children at an early stage, prompting them to see the doctor. This might also be supported by the fact that there was no difference in age of onset between the two groups and the duration of illness (the period from disease onset to the visit to the clinic) was shortened in the After group in the current study.

The EDI profile of patients visiting our clinic before and after the first state of emergency significantly differed. The higher rate of AN-R may explain the lower bulimia subscale score among the patients who visited the clinic after the first state of emergency. The significantly higher scores on maturity fears and significantly lower scores on ineffectiveness, as well as the lower tendency of drive for thinness and lack of interoceptive awareness, could be due to patients of younger age visiting after the first state of emergency[22]. Therefore, the findings imply that therapeutic intervention focusing on maturity fear may be beneficial for patients with COVID-19 pandemic lockdown-associated EDs.

A strength of the study is that we could, in a timely manner, focus on COVID-19 using a relatively large sample size to determine the characteristics of post-pandemic ED patients. The study also has several limitations. First, it is retrospective and cross-sectional, thus causality can only be inferred. Future longitudinal studies are required for further verification of the findings. Second, the patients were all female. Thus, the current results cannot be applied to male patients. Finally, because of the defensive nature of AN patients, the current study relied on self-report measures, and patients may not have provided accurate responses.

## Conclusions

Confinement at home associated with the COVID-19 pandemic and a related close relationship between parents and children may have influenced the decreased age of ED patients at their initial consultation. Treatment interventions should consider differences in the clinical features of EDs.

## Data Availability

The datasets analyzed in the current study are available from the corresponding author upon reasonable request.

## Abbreviations

AN: anorexia nervosa
AN-R: restricting type of anorexia nervosa
AN-BP: binge eating / purging type of anorexia nervosa
BN: bulimia nervosa
ARFID: avoidant / restrictive food intake disorder
BED: binge eating disorder
OSFED: other specified feeding and eating disorder
UFED: unspecified feeding or eating disorder
EDI: Eating Disorder Inventory
PBI: Parental Bonding Instrument
BMI: Body mass index
